# Development of physical literacy in school children and adolescents through the intervention of a multicomponent program of physical activity in the natural environment

**DOI:** 10.3389/fspor.2025.1686706

**Published:** 2026-03-13

**Authors:** Antonio Castillo-Paredes, Carmen Galán-Arroyo, Jorge Rojo-Ramos

**Affiliations:** 1 Grupo AFySE, Investigación en Actividad Física y Salud Escolar, Escuela de Pedagogía en Educación Física, Facultad de Educación, Universidad de Las Américas, Santiago, Chile; 2 BioẼrgon Research Group, Faculty of Sport Sciences, University of Extremadura, Cáceres, Spain; 3 Promoting a Healthy Society Research Group (PHeSO), Faculty of Sport Sciences, University of Extremadura, Cáceres, Spain

**Keywords:** physical literacy, physical activity, natural environment, adventure education, school

## Abstract

**Background/objectives:**

Physical literacy (PL) is foundational for integrated education. Through physical movement, it is possible to develop skills, knowledge, attitudes, and confidence, producing improvements at the physical, mental, emotional, and social levels. This study aimed to analyze the effects of an intervention based on a multicomponent physical activity (PA) program in the natural environment on the development of PL domains in children and adolescents.

**Methods:**

This randomized controlled trial included an intervention group (*n* = 102) and a control group (*n* = 104). Over six consecutive days, the intervention group participated in a multicomponent outdoor PA program, while the control group continued their regular Physical Education classes. The analyses were performed using repeated-measures ANOVA, with eta squared (*η*^2^p) as the effect size indicator.

**Results:**

The intervention group showed significant improvements in Factor 1, sense of self and self-confidence (*p* < 0.001); Factor 2, self-expression and communication with others (*p* < 0.001); and PL (*p* < 0.001).

**Conclusions:**

Participation in a multicomponent outdoor PA program can effectively promote the development of PL, sense of self and self-confidence, and self-expression and communication with others.

## 1 Introduction

Physical literacy (PL) is of relevant importance to educate individuals through physical movement, as it allows the comprehensive development of skills, knowledge, attitudes, and confidence, providing substantial experiences that contribute to physical, mental, emotional, and social development through physical activity (PA) (1, 2). In this sense, PA today is not only associated with body movements that expend energy but also with motivations, desires, or needs that people have to participate in activities that promote personal or social development, oriented toward health, recreation, studies, wellness, and work, among others (3). In addition, exercise has been shown to benefit the motor and social development of school-age children (4, 5). However, currently, there is little scientific evidence on the design and development of specific programs for environments other than schools, such as natural environments (6, 7), which comprehensively promote the development of PL.

On the other hand, health benefits have been observed in people who engage in PA in natural environments (8). Similarly, the availability of natural spaces and environments is key to promoting PA in adolescents, potentially inducing the development of healthy behaviors (9). Moreover, the availability of these environments is beneficial for the development of children and adolescents (10). However, the school environment and the relationship between home and school influence PA practice (11).

In line with the above, the pedagogical model proposed by Baena Extremera (12) states that through adventure education, emotional, personal, and social competencies can be developed through the practice of PA in nature, because it can promote significant learning through this direct and reflective experience by the students and therefore the possibility of transferring this learning to various contexts, without losing its link to the objectives of this research. From this point of view, the development of programs and interventions associated with the practice of physical activity in the natural environment (PANE) has a significant pedagogical value, which, when integrated into the school environment, promotes experiential learning that positively affects the development of personal and social skills in students, such as autonomy (decision-making), empathy (valuing emotions and personal and collective needs), and cooperation (solving conflicts and personal and collective problems) (13), which are key skills for the development of daily life activities. In addition, because this approach is based on adventure pedagogy, it highlights the relevance of the design and development of planned interventions that allow the promotion of meaningful learning through challenges, reflection, and interaction in the natural environment, which promotes the integral development of students.

Considering the fundamental principles of the pedagogical model (12) and adventure pedagogy, both focus on the development of fundamental competencies, which highlight the educational value acquired through the practice and participation of PANE. This research, based on this pedagogical approach, allows, through experiential learning and subsequent reflection, the promotion of significant learning acquired through movement, which enables the development of sense of self and self-confidence, self-expression and communication with others, knowledge and understanding, and physical literacy.

Regarding adventure pedagogy, Caballero Blanco (13) states that, due to its characteristics, the proposed activities should consider the challenge, uncertainty, and interaction with the environment, which are considered relevant aspects to strengthen the students' comprehensive learning. It is for this reason that the present intervention proposal is in accordance with the exposed literature, because, for the fundamental development of the PL, the challenges for its construction were considered, which translates into self-knowledge, effective communication and understanding of the environment, which objectively translates into achieving the effective transition of the acquired learning in various educational, personal and social contexts. The present research hypothesizes that the implementation of a multicomponent program of physical activity in the natural environment and based on the adventure pedagogy of Caballero Blanco (13) and the pedagogical model of Baena Extremera (12), will allow the improvement in the dimensions of physical literacy in children and adolescents. It is for this reason that the objective of this research is to analyze the effects of an intervention based on a multicomponent program of physical activity in the natural environment for the development of the domains of physical literacy in children and adolescents.

## 2 Materials and methods

### 2.1 Design and ethics

The present pilot study was a randomized controlled trial (14, 15), consisting of two groups (intervention group and control group). Informed consent was obtained from the parents or legal guardians of all participants prior to their involvement in the intervention and the application of the study instruments.

The confidentiality of the participants' information was always guaranteed, and all data were collected anonymously. This research was approved by the Ethics Committee of the EDUCA platform for excellence in education research with authorization code 20/2024, in accordance with the Declaration of Helsinki (16) and the Singapore Declaration (17).

### 2.2 Sample

The sample was selected in a probabilistic manner, ensuring that all participants had the same probability of participating. This approach enhances representativeness, reduces researcher bias, and ensures transparency and reproducibility (18). A total of 206 participants (109 girls and 97 boys) were divided into two groups: a control group (*n* = 104; M = 13.72 years) and an intervention group (*n* = 102; M = 12.33 years).

### 2.3 Selection procedure and criteria

To carry out the study, several public and private schools in the Autonomous Community of Extremadura were contacted and invited to participate in the research. The inclusion criteria required participants to be enrolled in Physical Education and to have no physical or psychological conditions that could prevent safe participation in physical activities in the natural environment. In this way, the safety and homogeneity of the sample were guaranteed, making it possible to obtain reliable and representative results on participation in outdoor physical activities.

Interested centers were presented with a detailed presentation of the physical activity intervention program in the natural environment, explaining its objectives, the structure of the multicomponent program, and the implications of participating. After confirming the willingness of the centers to collaborate, a definitive list was drawn up. Subsequently, a random draw was conducted to select two integrated educational centers that offered Physical Education across both the final cycle of Primary Education (5th–6th grades) and the first cycle of Secondary Education [1st–4th Educación Secundaria Obligatoria (ESO)] within the same campus. One center was then randomly assigned to the intervention group and the other to the control group. This procedure ensured an impartial selection and a homogeneous age range (approximately 12–14 years) while maintaining educational diversity within integrated institutions.

The selected educational centers are considered integrated centers, a type of institution common among subsidized private schools that teach primary and secondary education in the Autonomous Community of Extremadura. This structure allowed for the inclusion of students in the last cycle of primary education and the first cycle of secondary education, maintaining a homogeneous age range (12–14 years).

Once the selection was made, both schools were contacted to formalize participation. Then, the samples were randomized, where one of the centers was assigned to the control group, where its students participated normally in the subject. The other center was assigned to the experimental group, where its students would participate in the intervention through the multicomponent program.

### 2.4 Multicomponent intervention program

**Figure 1:**
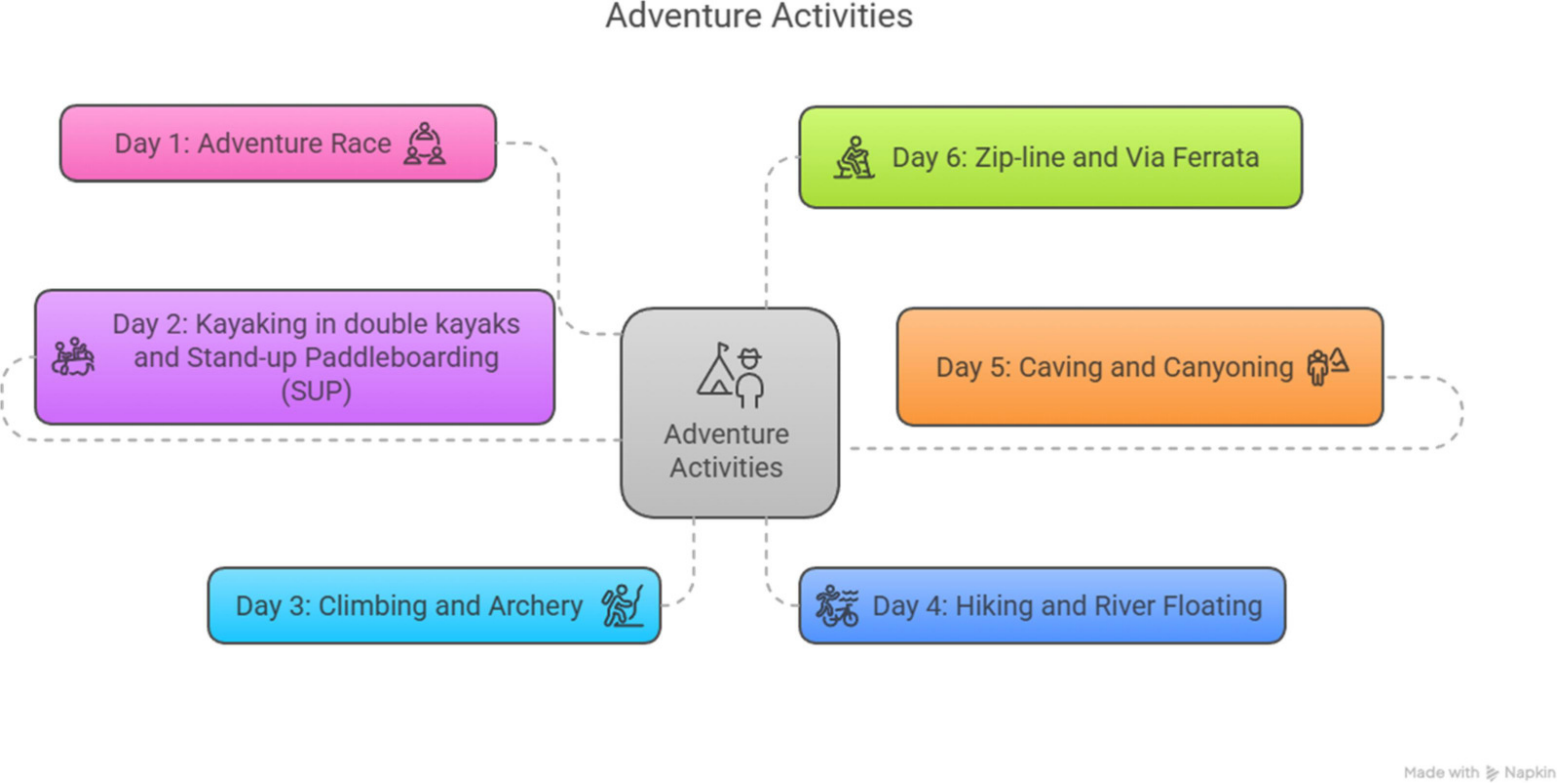
Diagram of the schedule of activities. Developed with Napkin.AI©.

The development of the intervention program was based on the pedagogical model of Adventure Education proposed by Baena Extremera (12). In the design, PANE was considered to promote sense of self and self-confidence, self-expression and communication with others, knowledge and understanding, and physical literacy. This program allowed the study participants to have meaningful experiences for the promotion of socioemotional learning, through a challenging process and personal improvement (Figure 1).

### 2.5 Design of the multicomponent intervention program

For the development of the research, a multicomponent intervention program was created, which lasted six consecutive days, with structured activities from beginning to end, and where the participants of the intervention were the PANE intervention group. The program had a structure of two sessions per day, during the morning and afternoon, with an approximate duration of 240 min (all days except Day 1 were 2 h in the morning and 2 h in the afternoon, and Day 1 was 4 h in the morning).

The moments of reflection, described as part of the pedagogical model of the intervention, were incorporated into the final minutes of each activity session as a structured space for feedback and analysis. Therefore, they did not extend the entirety of the scheduled sessions.

These reflection sessions were conducted with all 51 participants at the end of each activity, guided by the technical staff and supported by the research team. The process followed a brief but structured format, focused on identifying emotions, challenges, and key learnings from the activity. Although time constraints and group size limited the depth of discussion, the reflection moments provided meaningful opportunities for awareness and collective analysis within the pedagogical model of the intervention.

The objective of the proposed activities exclusively created for the research consisted of explicitly working on the variables studied, such as sense of self and self-confidence, self-expression and communication with others, and knowledge and understanding as transversal pedagogical themes. To promote significant learning and integral development of the participants, it was considered that these activities should be challenging and developed in a safe environment for the work of the group activities; thus, the integration of the physical and socioemotional components was present in the course of the activities proposed in the program (Table 1).

**Table 1:** Chronogram of activities.

Days	Activities
Day 1	Adventure Race (Morning)
Day 2	Kayaking in double kayaks
	Stand-up paddleboarding (SUP)
Day 3	Climbing
	Archery
Day 4	Hiking
	River Floating
Day 5	Caving
	Canyoning
Day 6	Zip-Line
	Via Ferrata

To ensure the viability and internal consistency of the intervention, all activities were carried out following standardized safety and organizational protocols. The program was implemented in collaboration with a company specializing in outdoor education and large group management, with extensive experience in organizing large-scale camps and numerous school trips in collaboration with educational agencies and tour operators. This company regularly manages programs with large numbers of children and adolescents, carrying out the same types of activities (such as climbing, via ferrata, zip-lining, caving, and canoeing) within similar time frames. Its procedures are highly systematized to adapt each activity to the time available, ensuring pedagogical quality and operational viability. All sessions were supervised by qualified instructors, including certified sports and mountain technicians and trainers from official sports technician schools and mountain federations. These professionals followed pre-established safety and logistics plans, ensuring the safe and efficient development of all activities during the intervention.

### 2.6 Procedures

#### 2.6.1 Research team and specialists

The program was designed, implemented, and supervised by professionals specialized in Physical Education and in the development of activities in the natural environment. In this way, the accompaniment and safety of the participants were guaranteed during the development of all the activities proposed in the program.

In addition, during the development of the activities, they were supervised by the research team, thus ensuring the proper implementation of the program, ensuring the timely and voluntary participation of all members, for the PANE intervention group, which participated in the multicomponent activities described, and the control group, which only participated in the scheduled assessments.

For the recruitment of participants, in the first phase, the research team contacted management and administrative personnel of the public and private educational centers of Extremadura by e-mail, where they were informed of the purpose of the program and its emphasis on PL, providing in detail the multicomponent activities to be developed, ensuring accompaniment, and providing the corresponding security measures for its implementation.

In the second phase, the schools that decided to participate provided the students with an informative dossier prepared with the research team, which included informed consent for parents or legal guardians and an enrollment form. Once all the informed consents were obtained, the participants were admitted to the multicomponent program intervention. Finally, participants were informed of the group to which they would be a part, either control or intervention.

To ensure methodological rigor and validity, cluster randomization was carried out by the educational center. In previous studies, this approach was used to avoid contamination between groups (19). Moreover, one of the educational centers was randomly assigned to the intervention group and the other to the control group using the “random” function in Microsoft Excel. This procedure was managed by a researcher who was not involved in the development of the intervention program.

The information was collected by specialized evaluators who were blinded to the assignment of participants. Similarly, the same instruments, instructions, and environmental conditions were used in pre- and post-intervention measurements to ensure consistency of the evaluations.

Prior to the start of the intervention, the research team received specific training to ensure the correct application of the program, as well as consistency in the execution of activities and compliance with safety and ethical protocols. In this way, measures sought to minimize possible biases, ensure the reproducibility of the study, and guarantee internal validity.

#### 2.6.2 Control group

As for the control group, a member of the research team visited the educational centers on the established dates, which coincided with the pre- and post-intervention periods of the intervention group. All participants (*n* = 104) in this group were evaluated according to the same criteria as the intervention group, where they were administered the instruments designed to measure the dimensions of PL (Factor 1, sense of self and self-confidence; Factor 2, self-expression and communication with others; Factor 3, knowledge and understanding).

During each evaluation session, the members of the control group were given a Tablet with access to the self-report questionnaire designed in Google Forms. With a duration of approximately 10 min, a researcher proceeded to read aloud the instructions and items of the questionnaire in order to ensure that all participants understood the questions and their respective responses to the components of the PL.

To ensure adequate supervision and safety during the implementation of the program, the 102 participants in the intervention group were randomly divided into two subgroups of 51 students through a group dynamic in all activities except for the multiadventure raid activity. Following the structure established in Table 1, both subgroups performed different activities simultaneously in the morning and afternoon sessions. For example, while Group A performed one of the activities (e.g., kayaking in double kayaks), Group B participated in the complementary activity (e.g., stand-up paddleboarding (SUP)), alternating in the next session so that everyone completed the full set of experiences over the 6 days.

In terms of technical supervision, there was one technician assigned for every six participants in the multiadventure raid (morning), climbing, river floating, caving, canyoning, and via ferrata activities and one technician for every eight participants in the SUP, kayaking in double kayaks, archery, hiking, and zip-line activities. All the technicians in charge had the professional qualifications required by Law 15/2015 of April 16, which regulates the practice of sports professions in Extremadura, thus ensuring compliance with safety and professional competence standards.

Students in the control group continued with their normal Physical Education classes. During the week in which the intervention was implemented, the control group continued with their regular Physical Education schedule, attending two weekly sessions of approximately 50 min each, as established in the school timetable.

Finally, the collected data were anonymized, where each participant was assigned a secret code assigned by the research team. This code, known by the participant, had to be included during the application of each instrument, thus ensuring traceability, privacy, confidentiality, and compliance with the ethical aspects of the study.

#### 2.6.3 Intervention group

The intervention group was composed of a total of 102 participants, who enrolled and participated voluntarily in the study. All participants were familiarized with the design of the multicomponent program to promote PL. As for the development of the program, it had the collaboration of Gecko Turismo Activo, who are leaders in the development of active tourism in the Jerte Valley in Extremadura.

The program was carried out in June 2024 and lasted 6 days in the natural environment of Extremadura. Its multicomponent design allowed the development of the dimensions of PL, such as sense of self and self-confidence, self-expression and communication with others, and knowledge and understanding of the environment. These activities were focused on experiential learning and learning through movement, structured to promote personal and social competencies that are key to the development of daily life activities.

The implementation of the program was led by professionals qualified in the development of outdoor physical activity, which ensured the accompaniment and complete safety of the participants. Each day of activity had two work sessions in the morning and afternoon, with a duration of 240 min. Among the activities developed were canyoning, canoeing, climbing, via ferrata, paddle surfing, hiking, caving, and archery. All these activities were designed to consider significant challenges that would favor a sense of self and self-confidence, self-expression and communication with others, and knowledge and understanding. Finally, each activity included instances of guided reflection to promote interaction between experiential learning and the development of PL for an integral conception.

### 2.7 Instruments

**Figure 2:**
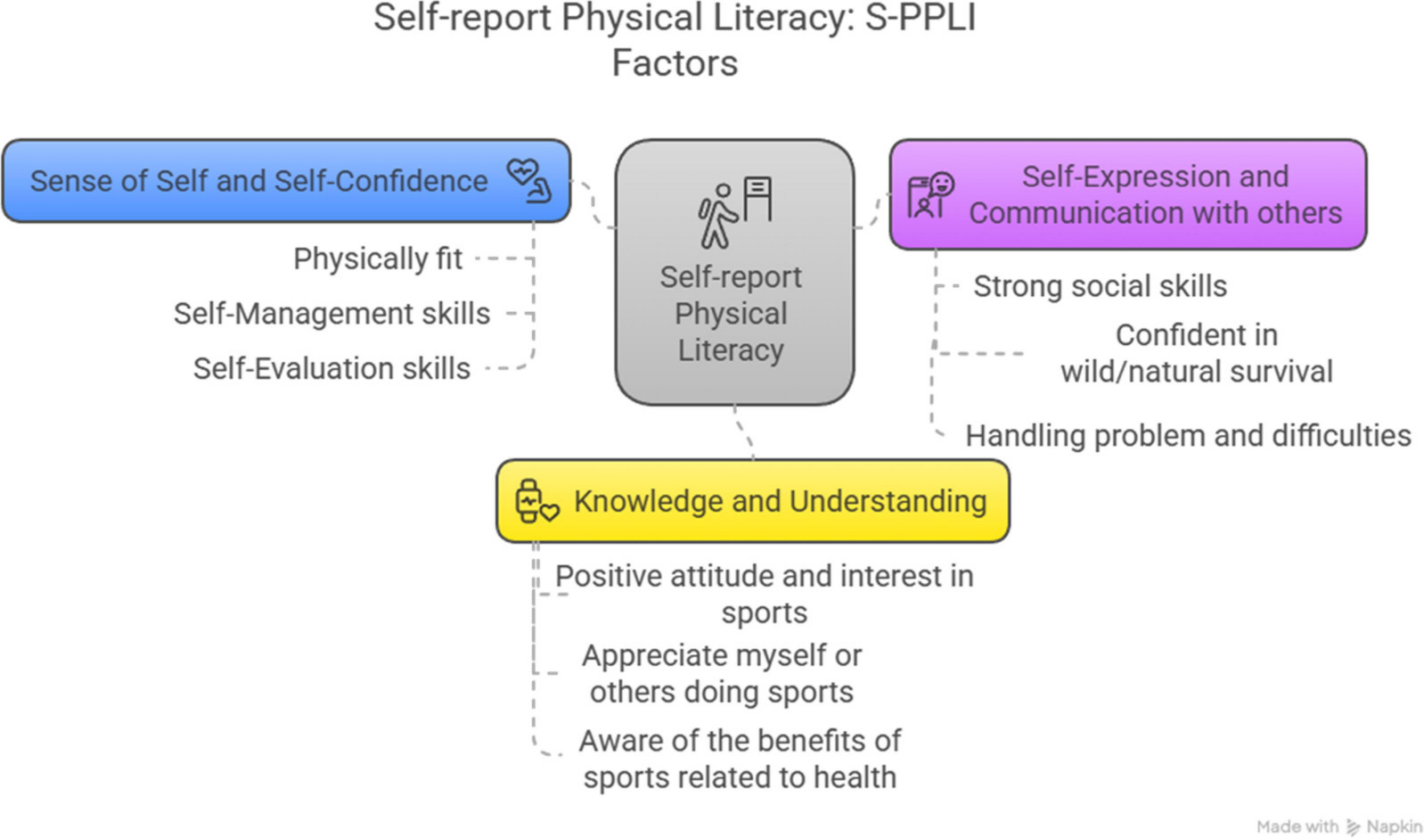
Physical literacy instrument for adolescents and its domains. Developed with Napkin.AI©.

For the development of this research, the Perceived Physical Literacy Instrument for Adolescents (S-PPLI) was used. This instrument has been adapted for Spanish adolescents (20); therefore, this instrument is reliable and valid for the school-age sample that was selected. This instrument was developed to measure knowledge related to physical competence, confidence, motivation, and understanding of PA for Physical Education teachers and then for adolescents (PPLI) (21, 22) (Figure 2).

On the other hand, this instrument was validated and adapted for Spanish adolescents (20), which is divided into three factors and subdivided into three items each, which are (a) sense of self and self-confidence (Factor 1), (b) self-expression and communication with others (Factor 2), and (c) knowledge and understanding (Factor 3). Each item has statements that have a score from 1 to 5, where 1 means “strongly disagree” and 5 “strongly agree” (Table 2).

**Table 2:** Factor and items of the physical literacy instrument for adolescents.

Factor	Item
Factor 1: sense of self and self-confidence	PL1 I am physically fit, in accordance with my age
	PL4 I possess self-management skills for fitness
	PL5 I possess self-evaluation skills for health
Factor 2: self-expression and communication with others	PL6 I have strong social skills
	PL7 I am confident in wild/natural survival
	PL8 I am capable of handling problems and difficulties
Factor 3: knowledge and understanding	PL2 I have a positive attitude and interest in sports
	PL3 I appreciate myself or others doing sports
	PL9 I am aware of the benefits of sports related to health

Own elaboration.

Finally, regarding the reliability and validity of the instrument, the authors stated the following Cronbach's alpha (*α*) rating of 0.87, and the confirmatory factor analysis showed that the responses fit adequately to the three-factor structure (ꭓ^2^ = 52.260, df = 24, *p* < 0.001, comparative fit index (CFI) = 0.976, root mean square error of approximation (RMSEA) = 0.057, standardized root mean square residual (SRMR) = 0.031).

### 2.8 Statistical analysis

For the analysis of the information, it was verified whether the distribution of the data corresponding to the quantitative variables complied with normality, for which the Kolmogorov–Smirnov test was used, confirming this assumption. In addition, the changes in the quantitative data corresponding to the intervention were examined through repeated-measures ANOVA over time, where the initial level of each of the study variables was considered as a covariate. Statistical analyses of the information were carried out by intention-to-treat (ITT) and per-protocol analyses. Between-group baseline differences were assessed with independent samples *t*-tests and within-group differences with paired samples *t*-tests.

The level of statistical significance was set at *p* < 0.05, and the effect size was calculated using partial eta squared (*η*^2^p), interpreted according to conventional benchmarks (small = 0.01, medium = 0.06, large = 0.14; 23). All data analyses were performed with the SPSS v.26 statistical package (IBM SPSS Statistics, version 26.0, IBM Corp, Armonk, NY, USA).

## 3 Results

Table 3 presents the characterization of the sample, including the distribution by sex, grade, and demographic location, as well as the means and standard deviations of age, height, weight, hours of daily physical activity, and hours of screen time per day for the control and experimental groups. These data provide an overview of the demographic and physical characteristics of the participants in both groups.

**Table 3:** Characterization of the sample (*N* = 206).

Variable	Category	*N*		%	
		Control	Intervention	Control	Intervention
Sex	Boys	40	57	38.5	55.9
	Girls	64	45	61.5	44.1
Course	5° grade	5	19	4.8	18.6
	6° grade	18	29	17.3	28.4
	1° ESO	16	17	15.4	16.7
	2° ESO	26	26	25	25.5
	3° ESO	11	9	10.6	8.8
	4° ESO	14	2	13.5	2
	1° Baccalaureate	14	0	13.5	0
Demographic location	Rural	44	56	42.3	54.9
	Urban	60	46	57.7	45.1
Variable		M		SD	
		Control	Intervention	Control	Intervention
Age		13.72	12.33	1.84	1.49
Height (m)		1.60	1.56	0.10	0.13
Weight (kg)		51.92	45.96	11.10	11.87

*N*, number; %, percentage; SD, standard deviation; M, mean; ESO, Educación Secundaria Obligatoria (in Spanish) (Compulsory Secondary Education).

Table 4 shows the means, standard deviations, independent samples *t*-tests, and repeated-measures ANOVA for each of the outcomes of the variables evaluated in the study. The intervention group showed significant intragroup improvements in variables such as Factor 1, sense of self and self-confidence (*p* < 0.001); Factor 2, self-expression and communication with others (*p* < 0.001); and physical literacy (*p* < 0.001) in the intervention group. Although for Factor 3, knowledge and understanding, in the control group (*p* 0.008), no significant interactions were observed.

**Table 4:** Means, standard deviations, independent sample tests, and repeated-measures ANOVA.

Variable	Intervention group				Control group				*F*-value	*p* inter	Effect size (*η*^2^p)
	*N*	M (SD) pre	M (SD) post	*p* intra	*N*	M (SD) pre	M (SD) post	*p* intra			
Factor 1: sense of self and self-confidence	90	3.60 (0.78)	3.90 (0.92)	<0.001	97	3.45 (0.73)	3.91 (0.81)	<0.001	1.873	0.173	0.010
Factor 2: self-expression and communication with others	90	1.14 (0.27)	3.61 (0.90)	<0.001	97	1.14 (0.26)	3.55 (0.85)	<0.001	0.516	0.473	0.003
Factor 3: knowledge and understanding	90	4.25 (0.87)	4.31 (0.91)	0.008	97	4.00 (1.04)	4.24 (0.74)	<0.001	1.406	0.237	0.007
Physical Literacy	90	3.83 (0.67)	4.05 (0.80)	<0.001	97	3.71 (0.68)	4.03 (0.65)	<0.001	1.211	0.273	0.007

The initial level of the participants in each of the variables studied was used as a covariate in the ANOVA.

Table 5 shows the Cronbach's alpha values for the different dimensions evaluated in the study, both globally and differentiated in the intervention and control groups, before and after the intervention. All the variables studied, except assertiveness and problem-solving obtained satisfactory values according to Nunnally and Bernstein (24).

**Table 5:** Internal consistency coefficients of each variable pre and post.

Variable	Global		Intervention group		Control group	
	Cronbach’s *α* pre	Cronbach’s *α* post	Cronbach’s *α* pre	Cronbach’s *α* post	Cronbach’s *α* pre	Cronbach’s *α* post
Factor 1: sense of self and self-confidence	0.789	0.734	0.799	0.722	0.760	0.750
Factor 2: self-expression and communication with others	0.754	0.702	0.771	0.743	0.745	0.651
Factor 3: knowledge and understanding	0.840	0.763	0.785	0.657	0.878	0.844
Physical literacy	0.846	0.872	0.835	0.884	0.856	0.856

## 4 Discussion

The present research aimed to analyze the effects of an intervention based on a multicomponent program of physical activity in the natural environment for the development of physical literacy domains in adolescents.

Regarding the results obtained, it was observed that there are significant pre- and post-differences for the group that participated in the multicomponent intervention, in the dimensions of sense of self and self-confidence, self-expression and communication with others, knowledge and understanding, and total physical literacy. In the study conducted by Choi et al. (25) in Hong Kong, adolescents noted that there is a significantly low association between PL and PA recreationally; however, they also noted that the dimensions sense of self and self-confidence, self-expression and communication with others, knowledge and understanding, and total physical literacy were positively related to PA. This may be related to the fact that those with higher total PL scores have a higher adherence to participate in programs and interventions that promote PA (26).

These results reflect that the intervention program had a positive impact on the promotion of physical literacy and its dimensions. Even if the effect sizes are small, this could be related to the fact that for each point increase in PL, and there is an increase in PA (27). On the other hand, it has been evidenced that improvements in the dimensions of the PL could reduce or prevent symptoms of depression, anxiety, and stress in the youth population (28), because an increase in the levels of these symptoms decreases the possibility of communicating, expressing oneself, making decisions, and emotional regulation (29–31).

On the other hand, although there are no significant results in some variables of the study, in self-expression and communication with others, intragroup improvements were obtained in both groups; therefore, the development of multicomponent programs contributes to the competencies and dimensions that are related to physical literacy.

In addition, the achievement of key results for sense of self and self-confidence is one of the best intragroup results. This may be related to the fact that the development of activities in natural environments requires the strengthening of self-efficacy and the perception of personal control (32). In addition, developing sports PA in a natural environment allows the acquisition of healthy behaviors, which allow improvements to health (33), among them, producing improvements in attention and working memory after a 15 min walk outdoors (34).

Another point of relevance is that the promotion of PA in these environments increases PA levels and participation to the extent that there is knowledge of these available spaces, allowing the possibility of reducing environmental or social barriers to obtaining these benefits (35). On the other hand, it has been evidenced that, currently, the practice of PA in natural environments has been increasing, because it promotes psychological health in adolescents and the young population (36). It has also been shown that those adolescents who are physically literate, along with their dimensions, have higher academic performance (37).

Another relevant point that favors our results may be related to differences in physical and psychological characteristics, growth, maturation, and motor development. This is because there is a decrease in physical activity during the transition from childhood to adolescence (38). From this point of view, the possibilities for participating in natural environments are varied and different from those that students are already accustomed to (39). Growth, maturation, and motor development are relevant for children and adolescents to participate in physical activity because the development of these variables leads to better health, prevention of exercise deficiency disorders, and improved neuromuscular control (40).

The results obtained in this multicomponent physical activity program in the natural environment have been significant in the areas mentioned above. This may be related to the restorative effects that children and adolescents experience when exposed to nature, which are linked to behavioral, emotional, social, and complex variables, allowing for improvement in these areas due to people's interaction with their environment, which is different from the educational center (41). Moreover, the possibility of interacting with nature and expert-guided work could be explained by the Hawthorne effect, which posits that people's behavior changes when they are aware that they are being observed and there are positive reinforcements for the achievement of activities (42). Similarly, our results could be mediated by self-determination theory, related to intrinsic and extrinsic motivation, because the development of the proposed activities allows for the satisfaction of autonomy, competence, motivation, and the social context among peers, enabling them to continuously seek new experiences and active challenges to develop and master themselves to achieve the objectives set (43).

Finally, although participation in programs that promote physical activity in natural environments is beneficial (44), it is not without certain limitations or barriers to its development and successful implementation. This is because distance, accessibility, neighborhood configuration, participants' level of physical activity, and programming affect how often people obtain the benefits experienced when engaging in physical activity in natural environments (8). The points mentioned above are key when considering a program with similar characteristics, which are beneficial for improving mental health, promoting physical activity, and improving physical health (45).

### 4.1 Limitations and strengths

Our study on physical literacy is not free of limitations and strengths. In terms of limitations, it is related to obtaining the sample, since the school population in Extremadura for the 2024–2025 cycle was 168,178 students in non-university education, which includes both primary and secondary; therefore, the size of our sample is smaller. Similarly, although the effect size on self-expression and communication with others is small, it was observed that in the changes reflected, there was a significant improvement in the skills in an integrated way in the development of physical literacy; however, extrapolation of the results should be taken with caution in larger samples, because although the sample selection was probabilistic, it did not reach 95%. This is because during the process, there were limitations on voluntary participation due to project deadlines, authorizations, and informed consents from the parents and/or legal guardians of the participants.

Finally, cluster randomization was performed, assigning entire schools to either the control or intervention group. This approach may have introduced a possible clustering effect that limits statistical independence.

In terms of strengths, this is one of the few studies that analyzes physical literacy and its dimensions after participating in a multicomponent program of physical activity in the natural environment, in a school population, guided and mediated by experts in activities in the natural environment and accompanied by a research team in the area. In addition, our findings highlight significant results for the strengthening of self-efficacy and the perception of individual control.

### 4.2 Future lines

Considering the results, limitations, and strengths, future work could extend the duration of the interventions. In this way, the results obtained in the dimensions of the PL could be enhanced through the participation of experiences that involve the practice of physical activity in the natural environment. In addition, the incorporation of extracurricular activities with a focus on the development of the dimensions of PL, related to corporal expression activities, conflict resolution, could strengthen the emotional and social competencies associated with PL, obtaining key results in the participants, which could be transferred to daily life activities or other educational environments.

Similarly, future research could consider a larger sample size and a multicenter, longitudinal, follow-up design that allows for the inclusion of other variables for objective and subjective measurement.

### 4.3 Practical implications

PL could be presented as a key integral approach for the development of its dimensions in people. This is because, through the practice of PA, individuals can develop a sense of self and self-confidence, self-expression and communication with others, knowledge, and understanding. PL goes beyond just keeping moving or being physically active. It enables the promotion of a sense of self and self-confidence, self-expression and communication with others, knowledge and understanding, understanding of the environment, and contribution to the emotional and social well-being of its participants, since interaction among peers and with the environment are key and essential elements for personal growth.

Finally, the integration of PL in the educational curriculum allows the integral development of people, enabling them to be able to face daily life challenges with greater autonomy, confidence, security, and adaptability, which are skills that are required for a society that is constantly changing.

## 5 Conclusions

In this study, the participation of a multicomponent program of physical activity in the natural environment for the development of physical literacy showed significant improvements in sense of self and self-confidence, self-expression and communication with others, and PL, highlighting the effectiveness in the development of the dimensions of physical literacy. Although the effect size is small, the work developed shows progress in the development of these programs, which could be included in various school contexts, extracurricular workshops, or recess interventions.

## Data Availability

The datasets presented in this article are not readily available because, for ethical reasons, the data are only available for justified reasons. These must be requested from the author by correspondence. Requests to access the datasets should be directed to JR-R, jorgerr@unex.es.
